# Multi-Criteria Decision Analysis in Food Safety Risk Management: The Case of Dioxins in Baltic Fish

**DOI:** 10.3390/foods11071059

**Published:** 2022-04-06

**Authors:** Beshir M. Ali, M. G. Andersson, B. H. P. van den Borne, M. Focker, H. J. van der Fels-Klerx

**Affiliations:** 1 Business Economics Group, Wageningen University & Research, 6706 KN Wageningen, The Netherlands; bart.vandenborne@wur.nl (B.H.P.v.d.B.); marlous.focker@wur.nl (M.F.); ine.vanderfels@wur.nl (H.J.v.d.F.-K.); 2 Section for Environment and Feed Hygiene, Department for Chemistry, Environment and Feed Hygiene, National Veterinary Institute (SVA), 75189 Uppsala, Sweden; gunnar.andersson@sva.se

**Keywords:** chemical, contamination, decision making, derogation, MCDA, trade-offs

## Abstract

The Swedish risk management case of Baltic fatty fishes, in which dioxin levels may be too high, is a typical multidimensional food safety decision problem involving public health, economic, environmental and socio-cultural aspects. To effectively address the dioxin food safety problem, the multiple dimensions and conflicting interests of stakeholders have to be considered systematically when evaluating competing risk management options. The objectives of this study were to illustrate the applicability of the Multi-Criteria Decision Analysis (MCDA) method for multidimensional food safety risk management problems, and to evaluate the Swedish dioxin risk management using MCDA. The results show that the MCDA method is indeed a relevant tool for modelling the multifactorial Swedish dioxin problem and for initiating discussions amongst stakeholders to increase the acceptance of chosen strategies. Abolishing the derogation from the European Commission’s maximum limits for the presence of dioxins in Swedish fish is the dominant strategy for risk assessors, whereas the preferences provided by the other stakeholders would suggest a continuation of the derogation without providing consumer information. However, the preferences of female consumers match with the 2011 decision of the Swedish government to ask for a derogation in combination with consumer information. The conclusion drawn from our MCDA analysis is comparable to the government’s decision that—given the gradual reduction in dioxin concentrations in Baltic fish—the decision to continue providing consumer information or not mainly depends on how risk managers balance the preferences of the different stakeholders.

## 1. Introduction

Food safety has increasingly become a public concern due to the occurrence of various food safety incidents in recent decades [1,2,3] and the increased media attention to food safety incidents [4]. Risk analysis, which comprises the three interrelated activities of risk assessment (RA), risk management (RM) and risk communication, is used to optimize food safety RM decisions [5]. RA and RM are often separated to ensure the scientific integrity of the RA and to avoid a potential interference of risk assessors on risk managers’ decisions. Besides the public health risks quantified in RA, risk managers often consider several socio-economic dimensions in food safety RM, including economic impacts, consumer acceptance, social sensitivity and environmental impacts. While (scientific) RA results are often very detailed and comprehensive, the other dimensions are seldomly quantified. Furthermore, it often remains unclear how risk managers balance the various dimensions amongst each other when making the final RM decision.

An illustrative example is the case of the RM of dioxins in Baltic fatty fish species in Sweden. Although wild-caught fatty fishes are (potentially) important sources of food in Sweden, the high dioxin concentration in these species has remained a public health concern. Dioxins and dioxin-like polychlorinated biphenyls (PCBs) are persistent environmental pollutants that are highly toxic and may cause cancer, reproductive and developmental problems, damage the immune system, and interfere with hormones [6]. Thermal and chemical processes (e.g., waste incineration, coal and wood combustion, pulp and paper production) are sources of dioxins and dioxin-like PCBs [7]. Both local sources and atmospheric deposition contribute to the Baltic Sea’s dioxin accumulation [8,9]. Since dioxins are fat-soluble compounds, they are absorbed by fatty fishes through the food web [10]. Dioxin accumulation in fatty fishes not only depends on the dioxin concentration in the sea but also on the age and size of the fish, population density, and feed availability [8,10,11].

In 2001, the European Commission (EC) set a maximum limit for the presence of dioxins in fish, with Commission Regulation No 2375/2001. The regulation was later replaced by Commission Regulation No 1881/2006, which set a maximum dioxins concentration limit of 8 pg/g for fish products [12]. In Sweden, unfortunately, the dioxin concentrations of fatty fishes often exceed the ML. Sweden (and Finland) opposed the MLs by stressing the socio-economic and cultural importance of fishing in the region. As a result, they were granted two separate temporary derogations from the limits for the periods of 2002 to 2006 and 2007 to 2011. The derogations allow the sale of wild-caught salmon, char, trout and Baltic herring (>17 cm) within its domestic market, while informing consumers about the health risks. To make a final decision regarding the derogation (i.e., to make it permanent or to abolish it), the Swedish government commissioned the National Food Agency (NFA) and the Board of Fisheries in 2009 to analyze the consequences for public health, and for the fishery and local businesses, respectively. 

The RA of the NFA [13] concluded that maintaining the derogation can, in the worst case, result in thousands of children and childbearing women facing the risk of exceeding the tolerable dioxin intake limit. The benefit assessment did not show the health benefits of eating Baltic fatty fishes that exceed the ML. Instead, the RA stated that the lost health benefits could be compensated by eating imported fish or fatty fish (with low levels of dioxins) from other regions of Sweden. On the other hand, based on an economic impact assessment, the Board of Fisheries [14] concluded that abolishing the derogation would most likely have large consequences for local fisheries, since fishermen depend on different species during different seasons and the loss of one target species may threaten the sustainability of the business. This in turn may impact local businesses that are associated with fishing. 

The decision to make the derogation permanent, in combination with consumer information, was described in a speech by the “State Secretary” (Ur N2016_05005), as a trade-off between the wish to maintain a sustainable fishing industry along the eastern coast and in the great lakes, and the public health risk. The expected consequences on fishing and local businesses, if the derogation would end, were considered to outweigh the public health risk. The decision of the government—which was against the RA recommendation—has been debated, specifically by scientists from the NFA (Gunnar Andersson, personal communication, 5 March 2021). Although several other aspects (e.g., preservation of coastal culture) have also been considered in the final decision, the lack of a formal RM report makes the decision non-transparent and, therefore, it is difficult to justify on what bases the decision has been made. 

Food safety RM problems are multidimensional in nature, as shown by the dioxin case. Risk managers have to consider these multiple dimensions and the conflicting interests of stakeholders when evaluating RM options to address the dioxin problem. Multi-Criteria Decision Analysis (MCDA) is a powerful methodology for evaluating and ranking competing options on multiple, often conflicting, criteria [15]. It is a method for objective and transparent RM using qualitative and/or quantitative data, while also considering stakeholders’ preferences. The method is officially recognized by the FAO and WHO for food safety risk analysis. It has been applied for RM in various fields (see Mardani et al. [16] for an overview), including the control of contagious animal diseases [17]. It has also been applied in hypothetical food safety RM cases [18,19], but to date, it has rarely been applied to real-life food safety RM procedures [20]. 

The objective of this study was to retrospectively evaluate the Swedish dioxin RM decision by applying MCDA. As a second objective, we illustrate the applicability of the MCDA method for multifactorial real-life food safety RM. The results of the MCDA procedure applied to the Swedish RM case of dioxin in Baltic fish confirm the multidimensional nature of the dioxin food safety RM problem, and the conflicting interests of stakeholders. The MCDA method is shown to be a suitable tool for balancing the multiple food safety dimensions, while considering stakeholders’ preferences in a transparent manner, by initiating discussions amongst stakeholders with conflicting interests.

## 2. Materials and Methods

Several MCDA methods have been proposed for selecting the best compromising options in multidimensional decision problems [15]. These methods can broadly be categorized into utility-based and outranking methods [21]. The main difference between MCDA methods is in the performance measurement over the criteria and the algorithms used to aggregate performance. Utility-based methods are based on the decision maker’s preferences that are defined using utility functions, as in the multi-attribute utility theory [22]. In contrast, outranking methods are based on pairwise comparisons of alternatives by utilizing the concept of dominance [23]. Option X dominates its alternative Y if X is at least as good as Y over all the criteria under consideration, and when X adequately outperforms Y on at least one criterion. Outranking methods allow the comparison of alternatives that are said to be ‘incomparable’ or ‘difficult to compare’ [23], and consider stakeholders’ preferences without defining utility functions. 

In this study the Preference Ranking Organization Method for Enrichment Evaluations (PROMETHEE) method was used [24]. PROMETHEE is a simple evaluation and an outranking method in ‘conception and application’, compared to other MCDA methods, [15] and has been widely applied due to its user-friendliness and mathematical properties [25]. PROMETHEE is suitable for food safety RM as it approximates real decision-making processes and it is easy to operationalize in a field to which it is unaccustomed [19]. Several steps have to be followed for implementing PROMETHEE in multidimensional decision problems [21]:

(1) Decision problem definition and stakeholder identification;
(2) Identification of RM options;
(3) Definition of criteria;
(4) Construction of performance matrix; and
(5) Producing the overall ranking.

The above five steps were followed for evaluation of the Swedish historical food safety RM problem of dioxins in Baltic fatty fish.

### 2.1. The Decision Problem and Stakeholder Identification

Fish is expected to be an integral part of a sustainable diet for ensuring food security in the face of climate change and the growing demand for animal food sources, since it is rich in protein, fatty acids, vitamins and minerals [26]. It is an important part of the Swedish diet, with an annual per capita consumption of 26.4 kg in 2016 [27]. Salmon and cod, mainly imported, are the two main consumed species in Sweden [28], but the consumption of Baltic salmon and cod is very low due to the limited supplies following the identification of high dioxin contents and the poor state of the Baltic Sea stocks [29,30]. In contrast, herring is one of the most abundant species in the Baltic Sea [11,29]. Currently, its use for food is very limited and the majority of the catches are used for feed [29]. There is, however, a growing shared interest among stakeholders to utilize Baltic fishes for food by managing the dioxin problem efficiently through integrated governance [31,32]. The reasons for this growing interest are that (i) fishermen would financially benefit from higher producer prices of Baltic herring for food than for feed; (ii) using herring for food would create new jobs along the fish value chain; (iii) the viability of coastal communities would improve; and (iv) wild-caught fish from the Baltic Sea is more environmentally sustainable than farmed and imported fish.

Despite the fact that the health benefits of consuming Baltic fatty fish outweigh the risks [33], the health risk perception of the dioxin problem has been negatively affecting the “fishing livelihood, the coastal culture, and the availability of fish products for consumers” [31]. Although dioxin levels still exceed the MLs for the presence of dioxins in fish, as defined by the EC (Regulation EC 1259/2011; Recommendation EC 2016/688), in some regions (e.g., Bothnian Bay and the Gulf of Bothnia) [8], the potential health risks associated with dioxins in Baltic fishes have been reduced by at least 50% in the last decade, due to the significant reductions in dioxin accumulation in the Baltic Sea since the 1970s [33]. If this declining trend continues, the reduction would result into dioxin levels in most Baltic fish species that are below the MLs in the near future. However, it would not result in human exposure to dioxins below the Tolerable Weekly Intake (TWI), recently suggested by EFSA of 2 pg TEQ/kg body weight [34]. 

In 2009, the Swedish government discussed making the derogation permanent or abolishing it, by considering the public health risk of consuming wild-caught fishes that exceed the ML and the socio-economic values of fishing in the region. Here, the problem is multidimensional, and several stakeholders with conflicting interests are involved. The selection of stakeholders is an essential step in MCDA. For the dioxin case, six stakeholder groups were identified: the Board of Agriculture, regional authorities, the NFA, fishermen, local businesses and consumers.

1. Board of Agriculture: this board represents risk managers at the state level, who are responsible for making decisions on measures to manage food safety (e.g., formulating rules and regulations) and for making the final decision regarding the derogation by considering the different criteria, and the political implications of the competing RM options.
2. Regional authorities: these are also risk managers, independent of the government, at regional level. In Sweden, regional authorities are responsible for the management of the great lakes (where fish catches from the lakes of Vänern and Vättern are considered as Baltic fish) and are involved in activities for improving the ecosystem of the great lakes.
3. NFA: risk assessors (public health authorities) are responsible for the scientific RA of the human health risk related to the intake of dioxins with consuming Baltic fatty fishes, especially for high-risk groups (children and childbearing women) and frequent consumers (recreational/sport fishers).
4. Fishermen: this group represents the interests of professional, anglers and other recreational, and landowner fishers. Following the confiscation of the right to sport fishing in the Baltic Sea and the great lakes in 1993 by the government, sport fishing has been free for everyone (without compensation to landowners). Household fishing with nets is also free on a limited scale. In rivers such as Torne Älv, “landowner fishing” is practiced. Both continuing and abolishing the derogation, and their associated governance practices, have consequences for the businesses of fishers.
5. Local businesses: this group represents businesses along the fish value chain (processing, handcrafting, tourism). Restriction on fishing (following the EU ML) may reduce the supply of raw fish for processing and affect tourist fishing. It may affect the turnover of businesses and may result in the loss of jobs. Moreover, fish is part of traditional dishes, which are also served to tourists. The availability of local food is one of the important factors for attracting tourists and for increasing experiential loyalty [35]. Smoked (salmonids and herring) fishes are traditional products in the coastal areas of Sweden. The fermented herring, made from Baltic herring, is a well-known traditional food product in the region.
6. Consumers: RM decisions affect consumers’ health, product choice, product quality and price. Some consumers might be concerned with the high dioxin levels in Baltic fatty fishes. However, they might not be willing to substitute them with other fish products or imported fish because of tradition, sustainability or culinary reasons.

### 2.2. Identification of Risk Management Options

In food safety, this PROMETHEE step refers to generating a list of potential RM options that can be applied to reduce the health risk related to consuming unsafe food, while also considering the socio-economic, cultural and ecological consequences of the proposed options. 

Broadly, there have been two approaches for managing the dioxin problem in the Baltic Sea region. The first approach aims at reducing the public health risks of dioxins and dioxin-like PCBs by setting an ML for the presence of these compounds in food and feed products (Regulation EC 1259/2011; Recommendation EC 2016/688). The second approach aims at controlling the release of dioxins and dioxin-like PCBs into the environment by setting stricter industry emission standards (e.g., Directive 2008/56/EC; Directive 2010/75/EU). We have defined the following three RM options for the Swedish dioxin case. 

***Option A: No Derogation***. This RM option refers to the abolishment of the derogation. Hence, Sweden follows the ML as set by the EC. Fishermen should subsequently sort Baltic herring by size, since large herrings (>17 cm) are not allowed for food anymore with this RM option. Moreover, authorities should recommend consumers to eat less Baltic fish in order not to exceed the TWI. As imported and farmed fish are the main sources of fish consumption in Sweden, Option A would likely further reduce the current limited use of Baltic fishes for food. 

***Option B: Derogation in combination with consumer information***. This option refers to a permanent derogation from the EC MLs in combination with consumer information (e.g., diet recommendations and recipes). A proactive governance is required to raise the use of Baltic fish for food through providing consumers with sufficient information about the fish species, sustainability, health risks and benefits, and recipes [36]. Option B is the strategy that Sweden follows currently. Authorities advise children and adults to eat fish of different varieties 2–3 times a week. Children and childbearing women are advised to limit consumption to 2–3 times per year, and other consumers to once a week for fish that may contain high levels of dioxins (e.g., Baltic herring). Authorities are required to continuously monitor dioxin levels and management practices for reporting to the EC. Both small and large herrings are available for human consumption within Sweden. 

***Option C: Derogation***. This option refers to a permanent derogation from the EC MLs, but without providing consumer information (e.g., diet recommendations and recipes). Authorities do not recommend eating less or more Baltic fishes. Continuous monitoring of dioxin levels is not required. Both small and large herrings are allowed for direct human consumption within Sweden. 

Management measures that aim at reducing the release of pollutants into the environment and improve the ecosystem of the Baltic Sea influence the relative performance of RM options. Thus, the three RM options were evaluated under two management scenarios. The first scenario (2021 case) refers to a business-as-usual (BAU) scenario, representing the situation today, where the level of pollutants stays constant, given the current management practices. According to Goherr [37], the current management practices are not effective to substantially improve the ecosystem of the Baltic Sea since “collaboration between governments and businesses is driven by objectives relating to regional competitiveness, job creation and economic growth, while environmental and social objectives are secondary”. As a result, “the state of the Baltic Sea ecosystems shows no signs of recovery, instead, problems relating to eutrophication and hazardous substances (e.g., dioxins) will prevail in the future”. The second scenario (2031 case) refers to a hypothetical sustainable management (SM) scenario, where sustainable practices are implemented during the next 10 years for reducing the release of pollutants and for improving the ecosystem of the Baltic Sea. In this scenario, the Baltic Sea countries and other stakeholders are expected to strictly implement the available guidelines and regulations for limiting the release of pollutants and to improve the ecosystem of the Baltic Sea. The sustainable practices are assumed to result in a 50% reduction in the potential health risks associated with dioxins and dioxin-like PCBs in Baltic fishes by 2031, as compared to the 2021 situation, by sustaining and fostering the reduction that has been observed in the last decade. There are, however, (direct and indirect) costs associated with the implementation of the sustainable practices, e.g., associated with investment in improved technologies, and reductions in the volume of catches due to restrictions. 

### 2.3. Criteria Definition

The definition of criteria, within the MCDA terminology, refers to the list of attributes that decision makers use for discriminating the various alternatives, as well as the scale of measurement for each criterion. Although the criteria defined highly depend on the problem and the values of decision makers, the list should be exhaustive, consistent, non-redundant, concise, complete and clear [38]. Following the EU’s impact assessment guidance [39], 14 potential criteria were adapted by Mazzocchi et al. [40] for food safety RM. However, as Mazzocchi et al. [40] indicated, some of these criteria are not mutually exclusive. Accordingly, we reduced the 14 criteria into eight in this study by: (i) merging overlapping criteria (e.g., combining firm competition, administrative burdens on businesses and conduct of businesses to form “operation of businesses”), and (ii) dropping less relevant criteria for the Swedish dioxin case. We expect little/no difference in the performances of the three RM options over the dropped criteria. The dropped criteria are innovation and research; international trade and third countries; macroeconomic environment; and positive and negative distributional effects. On the other hand, we have split public health into health risk and benefit, as health benefit is as important as the risk in this case study, as described below. Moreover, ‘culture’ is included as a criterion, since it is one of the relevant criteria in the Swedish dioxin RM case. Each criterion is described as follows. 

***Public health risk and benefit***. The high dioxin levels in Baltic fishes pose health risks to consumers, especially for high-risk groups such as children and childbearing women. For example, a 2007 risk assessment result showed that about 5% of 17–40 years old women exceeded the then tolerable intake of 2 pg/kg body weight/DAY [41]. Here it is important to note that this assessment was conducted in 2007, when dioxin levels were high and the tolerable intake was also higher. However, since 2018 the tolerable intake has been significantly reduced to 2 pg/kg body weight/WEEK, which is 7 times less than the level in 2007. This decrease in the tolerable intake has an impact on the risk assessment, where it increases the likelihood that a consumer exceeds the tolerable intake. However, Baltic fatty fishes are important sources of food as they are rich in omega-3, vitamin D and other micronutrients. Omega-3 fatty acids are useful for cardiovascular disease (heart disease and stroke) prevention, the modulation of immune and inflammatory responses, and the prevention of malignancies [42]. The health risks and benefits may vary across the three RM options and the two scenarios. 

***Operation of businesses***. Food safety RM decisions may affect the operations of businesses (e.g., fishermen and other businesses in the fish value chain), for example, by imposing additional administrative, adjustment, compliance or transaction costs. The different RM options may affect the conduct and competitiveness of businesses. This criterion consists of the following indicators: administrative burdens on businesses; additional adjustment, compliance and transaction costs; cost or availability of essential inputs; creation of barriers for new suppliers and service providers; trade barriers; facilitation of anti-competitive behavior or the emergence of monopolies; market segmentation; global competitive position of EU firms; access to finance; investment cycle; stricter regulation of the conduct of a particular business; new or closing down of businesses; and products or businesses treated differently. For the Swedish dioxin case, the restriction on fishing in the Baltic Sea and the great lakes (Option A) may impact the businesses of fishermen and other local businesses along the fish value chain. 

***Consumer acceptance***. Food safety RM decisions affect consumers’ health, product choice, product quality and price. The success of any RM option depends on the perception of and its acceptance by consumers. This criterion consists of indicators such as prices; organoleptic characteristics (taste, smell, sight, touch); consumer choice; consumer preferences; and the provision of affiliated public goods. In the Swedish dioxin case, consumers might be concerned with the high dioxin concentration in Baltic fatty fishes. However, they might not be willing to substitute these fishes (e.g., the inexpensive Baltic herring local food) with imported fish or other products. 

***Burden on public authorities***. This refers to budgetary consequences and administrative burden on public authorities, associated with RM decisions. Different food safety RM decisions may result in different administrative burdens and may have different budgetary consequences for (national, regional and local) public authorities. For the Swedish dioxin case, the level of authorities’ involvement varies among the three RM options, which has different implications for the budget and administrative burden on authorities. The provision of consumer information (Option B) may require extra budget and exert more administrative burden than the “derogation” (Option C).

***Employment***. Food safety RM decisions may directly and/or indirectly facilitate the creation of new jobs or may result in a loss of jobs. This criterion captures impacts on job creation/loss of jobs and particular professions, groups of workers or self-employed persons. For the Swedish dioxin case, for example, the use of Baltic herring for food (Option B and C) would create new jobs along the fish value chain [31]. On the other hand, the restriction on fishing in the Baltic Sea and the great lakes (Option A) may result in the extinction of traditional foods and handcrafts that are associated with fishing. The loss of these foods and handcrafts lead to a loss of jobs. 

***Environment/sustainability***. Protein production, mainly from animal sources, is known for causing environmental impacts through the release of pollutants, and the use of scarce resources such as fossil fuel and arable land. The use of Baltic herring and salmon for direct human consumption is more environmentally sustainable than farmed and imported fish, and other climate-unfriendly food sources such as beef [26]. The use of Baltic herring for food has several environmental benefits, since it has a higher yield and because the catches remove nutrients from the sea [33]. Emissions of greenhouse gases, eutrophication potential and energy use can be used as indicators for this criterion. For the dioxin in fish case, for example, the restriction in the use of Baltic fish for food (Option A) may result in the use of environmental-unfriendly products, such as imported/farmed fish or livestock products, for obtaining the same amount of protein compared to Option C.

***Culture***. Food safety RM decisions may have an impact on the preservation of cultural heritage, cultural diversity, and citizens’ access to cultural resources and participation in cultural manifestations. Baltic herring and salmon have been an integral part of coastal culture and food traditions in the region [31]. The preservation of coastal culture was also one of the government’s justifications for making the derogation permanent. One of the important concerns was that a ban on fishing would end traditions that go back hundreds of years. Losing fishing in the coastal areas may be emotionally hard for the local communities. The restriction on fishing in the region may result in the extinction of traditional foods (fermented herring and smoked fish) and handcrafts that have been associated with fishing for centuries.

### 2.4. Construction of Performance Matrix

The matrix captures the performance of each RM option over each criterion, as well as the decision maker’s preference for how an alternative should perform in order for it to be preferred over its alternative, and the preference/criteria weights. In PROMETHEE, preference functions are used for conducting a pairwise comparison [24], and they enable decisions to be made on how much better an option has to perform in order for it to be considered superior to another option. For a *U-shape* preference function, the preference for option *X* over *Y* can be given by:

(1) $$P_{j}\left(X,Y\right)=\{\begin{array}{c} 1,\;\;if\;g_{j}\left(X\right)-g_{j}\left(Y\right)>q_{j}\\ 0,\;\;\;if\;g_{j}\left(X\right)-g_{j}\left(Y\right)\leq q_{j} \end{array}$$

where $P_{j}\left(X,Y\right)$ refers to the preference for option *X* against option *Y* on criteria *j*; $g_{j}\left(X\right)$ and $g_{j}\left(Y\right)$ refer to the performance scores of options *X* and option *Y* on criteria *j*, respectively; and the parameter *q* refers to an indifference threshold. The indifference threshold *q* describes the point up to which a difference in performance between the two options does not make any difference to the decision maker. It indicates the minimum required difference between two options on a specific criterion in order for the better performing option to be regarded as being preferred over its alternative. In the present study, we assumed a “*U-shape*” preference function for all stakeholder groups, with an indifference threshold of zero. 

Although PROMETHEE allows the integration of both quantitative and qualitative data, this case study relied on only qualitative data for measuring the performance of the RM options over the eight criteria, due to a lack of quantitative data. During the finalization of this paper, Tuomisto et al. [33] published the results from a quantitative health risk–benefit assessment for Baltic fishes. However, the scenarios they used for calculating the risks and benefits differ from our RM options. We used a 6-point scale (no impact, very low, low, medium, high, and very high impact) qualitative scoring, using Expert Knowledge Elicitation via questionnaires. The 6-point scale rating, with the inclusion of ‘no impact’, avoids the shortcoming of the 5-point Likert scale, which often does not cover the no impact option. Moreover, a ‘medium impact’ is not distinguished from a ‘neutral view’ in the 5-point Likert scale (i.e., score 3). The experts who were consulted for the scoring are: (i) one expert from the Risk-benefit Management and Environment Team of the NFA (*Expert A*), (ii) four experts from the Fishery Unit of the Board of Agriculture (*Expert B*), (iii) one expert from the Ecosystems and Environment Research Programme of the University of Helsinki (*Expert C*), and (iv) four experts from the County Administration Board (regional authorities) who have experience and expertise in the management and ecosystem of lake Vättern (*Expert D*). The (four) experts from the Board of Agriculture scored the RM options together as a group (i.e., individual scores of the four experts were not obtained), referred to as *Expert B*. This also applies for the experts from the regional authorities, referred to as *Expert D*. For the criteria that were scored by more than one expert, the averages of the scores were used (Table 1). For example, for the criterion *operation of businesses,* which was scored by *Expert B, C* and *D*, the average of the group score of *Expert B*, the individual score of *Expert C* and the group score of *Expert D* were used. Table 1 presents the performance matrix. The scores show the conflicting performance of the RM options over the different criteria. For example, *Option A* and *Option C* are rated to cause, respectively, a very low and a very high health risk, regardless of the management of the Baltic Sea. In contrast, *Option A* is rated to cause a (very) high negative impact on culture/tradition and environment/sustainability, while *Option C* is not expected to cause any negative impact on these criteria. 

The preference weights (criteria weights representing the relative importance of each criterion to the different stakeholder groups) are obtained from representatives of each stakeholder group using a questionnaire. For the NFA and the Board of Agriculture and Regional authorities, preference weights were elicited from *Expert A*, *Expert B* (as a group) and *Expert D* (as a group), respectively. For Fishermen and Local Businesses, they were obtained, respectively, from a representative for a fishermen’s organization and a small business operating in Bothnian Bay. Each representative was requested to provide preference weights by dividing 100 points among the eight criteria. For consumers, the weights were obtained from Swedish fish consumers using an online survey. The consent form regarding the ethical aspects of the survey (data handling, privacy and potential risks to respondents) was approved by the Social Sciences Ethics Committee of Wageningen University. The survey was advertised on Facebook with a paid post. Responses were obtained from a total of 307 respondents, of whom 185 were actual and potential wild-caught fatty fish consumers. The average of the weights elicited from these 185 consumers is used for the analysis. The preference weight of the consumers is disaggregated per consumer group: urban vs rural, and female vs male. Table 2 summarizes the preference weights. The weights show the conflicting preferences of the stakeholders. For example, the NFA strongly focuses on health risks (80%), whereas Local Businesses focus on operation of businesses (50%) and health benefits (30%). Consumers have almost equal preferences for health risks (27%) and environment/sustainability (24%).

### 2.5. Ranking of Risk Management Options

The final step in the MCDA process is ranking the RM options. Decision makers aim at minimizing risks and impacts, while benefits and positive scores are maximized (Table 1). In the pairwise comparison, any positive difference in the scores for two alternatives results in a higher ranking for the alternative with the higher score on that criterion (Equation (2)). The degree of preference for option *X* against option *Y* can be calculated as:

(2) $$\pi \left(X,Y\right)=\sum_{j=1}^{k}w_{j}P_{j}\left(X,Y\right)$$

where π(X,Y) is the degree of preference for option *X* over option *Y*, $w_{j}$ refers to the preference weight for criterion j (Table 2) and $P_{j}\left(X,Y\right)$ is the preference for option *X* over option *Y* on criteria *j*. 

In PROMETHEE, the overall ranking is based on the aggregation of all criteria with a weight assigned to each criterion, and the calculation of a net outranking flow for each RM option. This calculation has two components: the degree to which an RM option outranks other RM options (positive flows) and the degree to which an RM option is outranked by other RM options (negative flows). Positive flow quantifies the degree to which an option is preferred over all other options (i.e., the degree to which it ‘dominates’ all others) and is calculated as the sum of all pairwise comparisons in favor of an option (Equation (3)). Negative flow quantifies the degree to which all other options are preferred over the option of interest (Equation (4)) and is calculated as the sum of all pairwise comparisons, where the option under consideration is the less preferred of the other options. The net flow (Equation (5)) is used to provide an approximate measure of the overall preference. The higher the net flow, the more preferred the RM option is. The calculation of flows for RM option *X* can be written as:

(3) $$\pi^{+}\left(X\right)=\sum \frac{\pi \left(X,Y\right)}{n-1}$$

(4) $$\pi^{-}\left(X\right)=\sum \frac{\pi \left(Y,X\right)}{n-1}$$

(5) $$net\;flow\left(X\right)=\pi^{+}\left(X\right)-\pi^{-}\left(X\right)$$

where $\pi^{+}\left(X\right)$ and $\pi^{-}\left(X\right)$ are positive and negative flows, respectively, and *n* is the number of RM options.

The Visual PROMETHEE-GAIA software (Version 1.4, Academic Edition, B. Mareschal, Bruxelles, Belgium) was used to calculate the positive, negative and net flows of the six RM options. The sensitivity of the rankings of the RM options to changes in the (average) preference weights of the four main criteria are reported using the *Visual Stability Interval* of the PROMETHEE-GAIA software.

## 3. Results

The results of the rankings of the RM options, based on the net flows, are presented in Table 3 for each of the six stakeholders. The negative scores of the net flows of Option A imply that the degree by which Option A is dominated by the other options is greater than the degree by which Option A dominates the other options (i.e., the negative flow of Option A is greater than its positive flow, Equations (3)–(5)). The highest and lowest net flow scores are, respectively, obtained for Option A (0.72) and Option C (−0.56) for the NFA. 

Under the BAU scenario, Option A (abolishing the derogation) is the best strategy, given the preference weights set by the NFA. This reflects that the NFA is very concerned with public health risk (80%), and the other criteria are not (or less) important in the evaluation of dioxin RM options for the NFA (Table 2). Given the preference weights set by the other stakeholder groups, Option C (making the derogation permanent without providing consumer information) is the best option for all but one stakeholder group. The rankings of the RM options for each stakeholder group under the SM scenario are similar to the rankings under the BAU scenario, except for fishermen, for whom Option B (derogation in combination with consumer information) is the dominant strategy. The scores of the net flows have, however, increased under the SM scenario compared to the BAU levels. The rankings of the RM options are similar for different groups of consumers, where Option C is the dominant strategy under both scenarios. However, looking at female consumers alone, Option B is the dominant strategy under both scenarios. 

**Figure 1:**
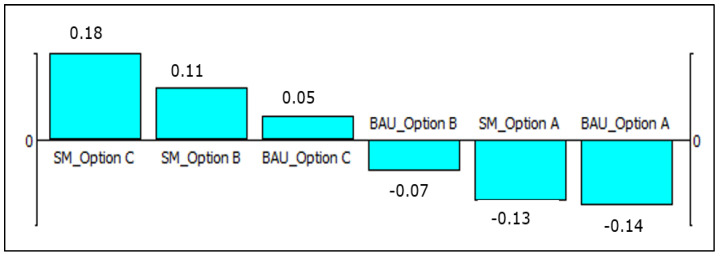
Net flows of the three risk management options under the business-as-usual and sustainable management scenarios. Note: The numbers refer to the net flows, which are derived using the average preference weights given in Table 2. Abbreviations: SM, Sustainable management scenario; BAU, Business-as-usual; Option A, No derogation; Option B, Derogation plus consumer information; Option C, Derogation.

The group (overall) ranking is presented in Figure 1. It is derived by calculating the net flow scores using the average of the preference weights of the six stakeholders (Table 2). (We also derived the group/overall ranking by scaling the net flows (Table 3) for each stakeholder group using the maximum net flow, i.e., by dividing the net flows with the absolute value of the maximum net flow. The results (not reported here) are similar with the ranking based on the average preference weights (Figure 1).) Under both scenarios, Option C is the best RM option, considering all stakeholder groups together and assuming that all stakeholder groups are assigned equal weight. Option C under the SM scenario is the global/overall best option from the six RM options under the two scenarios.

**Figure 2:**
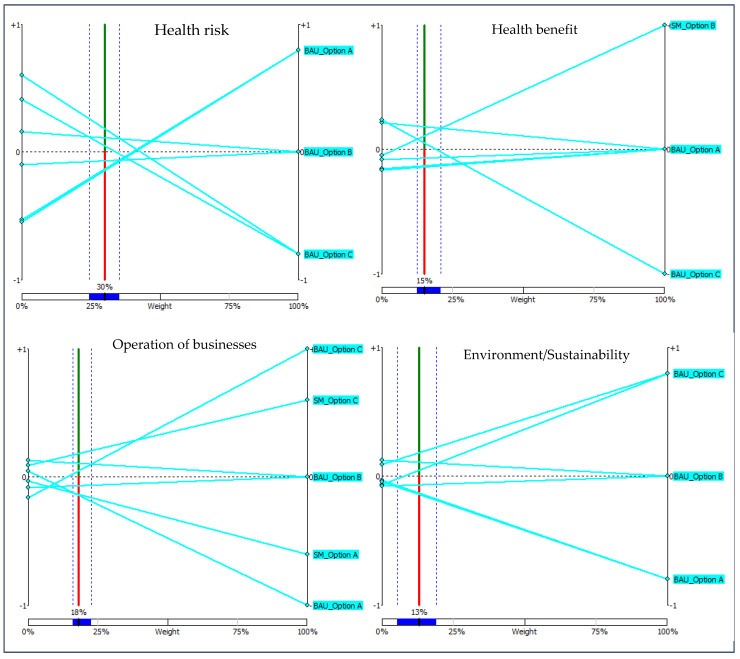
Visual Stability Interval of the four most valued preference weights (based on the average of the preference weights of the six stakeholder groups). Abbreviations: SM, Sustainable management scenario; BAU, Business-as-usual; Option A, No derogation; Option B, Derogation plus consumer information; Option C, Derogation.

The results of the sensitivity analyses are presented in Figure 2. On average, the six stakeholder groups consider public health risk (30%) and benefit (15%), operation of businesses (18%) and environment (13%) as the most important criteria for evaluating the RM options (Table 2). The overall/group ranking that is presented in Figure 1 will not change if the weight for public health risk remains between 24% and 35% (although the values of the net flow scores may change). Similarly, the ranking will not be affected if the weight for operation of businesses stays between 16% and 23%. Of these four criteria, operation of businesses has the narrowest stability interval, implying that the overall ranking is sensitive to the change in the weight of this criterion (Figure 2). 

## 4. Discussion

The results of the MCDA procedure applied to the Swedish RM case of dioxin in Baltic fish confirm the multidimensional nature of the dioxin food safety RM problem and the conflicting interests of stakeholders. The MCDA method is shown to be a suitable tool for balancing the multiple food safety dimensions while considering stakeholders’ preferences in a transparent manner. Although the MCDA method is officially recognized by the FAO and WHO for food safety RM, to date, it has not been applied to real food safety RM procedures. It has only been applied in several hypothetical food safety RM cases [18,19,20].

Tuomisto et al. [33] conducted a quantitative health risk–benefit assessment for Baltic herring and salmon in Sweden (and Finland, Denmark and Estonia). The results showed that the health benefits of eating Baltic fish outweigh the risks, even for the high-risk consumer groups, and they argue that “a more relevant value-based policy discussion rather than research is needed to clarify official recommendations related to dioxins in fish” [33]. The results from our case study strengthen the conclusion that values rather than scientific data determine which RM option comes out as the best. Whereas abolishing the derogation is shown to be the dominant strategy for the NFA, the weights assigned by the other stakeholder groups point in favor of making it permanent without providing consumer information. Moreover, the results showed that female consumers prefer the derogation in combination with consumer information (Option B), unlike the male consumers who prefer the derogation without consumer information (Option C). Female respondents attached a higher (36%) preference weight to the health risk criterion than male respondents (24%) (Table 2). This might be due to the fact that women (and children) belong to the high-risk consumer group for an increased level of dioxins in wild-caught fatty fishes [13] and due to the NFA’s information campaigns against Baltic fatty fish consumption, focused on high-risk consumer groups. 

We have used the average of the preference weights of the six stakeholder groups (Table 2) when deriving the group/overall ranking of the six RM options, that is presented in Figure 1. Ideally, this ranking should have been derived using agreed preference weights among the six stakeholder groups, where all the stakeholders discuss and reach consensus on group preference weights for the different criteria, e.g., by using the Delphi approach. In this case study, it is shown that Option C (derogation) under the SM scenario is the global/overall best RM option from the six competing options. This implies that if the dioxin levels continue to go down, as in the SM scenario, the best RM option would be to make the derogation permanent without targeted information campaigns, and thus indirectly promote an increased consumption of fatty fishes. 

Since all the stakeholder groups get the same weight and five of the six stakeholders prefer the continuity of the derogation (Table 3), Option C becomes the overall best RM option. However, this option has the lowest (worst) net flow for the NFA (−0.56 under the BAU and −0.44 under the SM scenarios). The NFA, focusing on public health risk, prefers the abolishment of the derogation, implying that the public health risk criterion is more important than the public health benefit and the other six socio-economic criteria. Communication amongst the six stakeholder groups is crucial to increase the effectiveness of chosen RM strategies. To this end, MCDA is a suitable method for engaging stakeholders in the decision-making process. The MCDA definition itself implies its suitability for facilitating communication amongst stakeholders. The National Research Council [43] defined it as “an approach used to systematically structure and model decision problems in multiple dimensions, with the goal of achieving a well-considered and -justified decision, and to provide a transparent explanation of the decision’s basis”. 

The similarity of the rankings of the RM options for all the stakeholder groups under the two scenarios (BAU and SM, Table 3) implies that a possible reduction in dioxin concentrations in the future will not affect the rankings of the considered RM options. However, the scores of the net flows have increased under the SM scenario compared to the scores under the BAU scenario for almost all stakeholder groups, since dioxins are less of a problem under the SM scenario (Table 3). This implies that improving the ecosystem of the Baltic Sea and the great lakes through sustainable management could contribute towards addressing the multidimensional dioxin problem.

The conclusions drawn from the dioxin RM case should be interpreted cautiously for the following reasons. First, qualitative data has been used; the reliability and robustness of the results of the present case study would increase if quantitative data were available (e.g., DALYs lost and prevented for public health risk and benefit under the different RM options). Second, some of the criteria were (qualitatively) scored by only one expert (e.g., public health risk and benefit by an expert from the NFA, and consumer acceptance and environment by an expert from the University of Helsinki), which might involve some degree of bias/subjectivity. Third, some of the stakeholder groups were represented by only one expert (e.g., the NFA) when eliciting the preference weights for the different criteria. However, it should be noted that it would not be relevant to ask for several answers from the NFA, as the ‘official’ answers may not be independent (as they work as a team). On the other hand, the number of small-scale business operators along the Baltic Sea coast is very low, and it may be very challenging to get respondents. For the fishermen, it might be relatively easy to get a response from a spokesperson, “interpreting” what s/he feels about the members’ thoughts. Hence, the use of a spokesperson’s response in the model may also introduce a bias. Despite these limitations related to the data used, the MCDA method is shown to be a relevant and transparent tool for modelling multifactorial food safety RM decision problems, while considering the interests of stakeholders.

In this study, we showed how stakeholders could participate in the decision-making process, at the later stage of the process for stating their preferences (e.g., after defining the problem, and formulating the alternatives and the criteria). However, the results of the MCDA might differ if stakeholders are involved in all the phases of the decision-making process (e.g., in defining the problem, formulating both the alternatives and the criteria). Participatory approaches that are used to draw stakeholders into the process of deciding between different alternatives, but not in the formulation of those alternatives, might not achieve the intended target of the policy. Therefore, a meaningful participation requires stakeholders’ engagement in all the phases of the process [44]. It would also be interesting for future research to investigate how the MCDA results differ according to the type of stakeholder engagement in the process (e.g., mediation vs negotiation) [45]. 

## 5. Conclusions

We illustrated the applicability of the MCDA method for balancing multiple dimensions in food safety RM decisions, while considering the conflicting interests of stakeholders, using the Swedish case of dioxins in Baltic fatty fish. The results show that the MCDA method is indeed a relevant and transparent tool for modelling the multifactorial dioxin problem and for initiating discussions amongst stakeholders with conflicting interests. The results also indicate that abolishing the derogation is the best RM option for the NFA, whereas the preferences provided by the other stakeholders would suggest making the derogation permanent without providing consumer information. The 2011 Swedish government’s decision to ask for a derogation with consumer information as an RM strategy agreed with the preferences of the most cautious and high-risk stakeholder group, the female consumers. Therefore, the conclusions drawn from this MCDA analysis are in line with the government’s decision that—given the gradual reduction in dioxin concentrations in Baltic fish—the decision to continue providing consumer information or not mainly depends on how risk managers balance the preferences of the different stakeholders.

## Figures and Tables

**Table 1:** Qualitative scoring of the Swedish dioxin in fish risk management options over the eight criteria.

					Business-as-Usual Scenario			Sustainable Management Scenario		
	Criteria	Scored by	Obj.	Pref. Fun. Threshold	Option A	Option B	Option C	Option A	Option B	Option C
**1**	Public health risk ^a^	*Expert A*	Min	U-shape; 0	1.00	2.00	5.00	1.00	2.00	5.00
**2**	Public health benefit ^b^	*Expert A*	Max	U-shape; 0	1.00	1.00	0.00	1.00	2.00	1.00
**3**	Operation of businesses ^a^	*Expert B, C, D*	Min	U-shape; 0	5.00	2.33	0.33	4.00	1.67	0.67
**4**	Burden on public authorities ^a^	*Expert A, B, D*	Min	U-shape; 0	2.00	1.67	0.33	2.33	2.00	1.33
**5**	Consumer acceptance ^c^	*Expert C*	Max	U-shape; 0	3.00	2.00	3.00	2.00	2.00	4.00
**6**	Employment ^a^	*Expert B, C, D*	Min	U-shape; 0	3.67	1.67	0.33	3.00	1.33	0.33
**7**	Environment ^a^	*Expert C*	Min	U-shape; 0	4.00	3.00	0.00	4.00	3.00	0.00
**8**	Culture ^a^	*Expert B, C*	Min	U-shape; 0	5.00	2.00	0.00	5.00	2.00	0.00

Abbreviations: Option A, No derogation; Option B, Derogation plus consumer information; Option C, Derogation. *Expert A, Expert B, Expert C* and *Expert D* refer to the experts from NFA, Board of Agriculture, University of Helsinki and Regional Authorities, respectively. ^a^ Measured on a 6-point scale from 0 = no negative impact to 5 = very high negative impact. ^b^ Measured on a 6-point scale from 0 = no benefit to 5 = very high benefit. ^c^ Measured on a 6-point scale from 0 = not acceptable to 5 = very high acceptance.

**Table 2:** Stakeholder preference weights (%) for the different criteria.

	BoA ^a^	RA ^a^	NFA ^b^	FM ^b^	LB ^b^	Consumers					Average ^c^
Criteria						All (n = 185)	Rural (n = 88)	Urban (n = 97)	Female (n = 50)	Male (n = 135)	
Public health risk	20	30	80	25	0	27	24	30	36	24	30
Public health benefit	10	10	0	25	30	17	20	15	16	18	15
Operation of businesses	15	20	0	10	50	10	12	8	8	11	18
Burden on public authorities	0	0	0	0	0	1	1	1	0	1	0
Consumer acceptance	5	0	20	10	10	6	6	5	4	6	9
Employment	10	5	0	20	0	6	6	4	5	5	7
Environment	15	25	0	10	5	24	20	28	23	25	13
Culture	25	10	0	0	5	10	11	9	7	11	8

Abbreviations: BoA, Board of Agriculture; RA, Regional authorities; NFA, National Food Agency; FM, Fishermen; LB, Local businesses. ^a^ The preference weights were obtained from four experts as a group (i.e., individual preference weights of the four experts were not obtained). ^b^ The preference weights from Fishermen and Local Businesses were obtained, respectively, from a representative of a fishermen’s organization and a small business operating in Bothnian Bay. ^c^ The average weight is derived as the average of the preference weights of the six stakeholder groups. For consumers, the overall preference weight is used.

**Table 3:** Net flows of risk management (RM) options per stakeholder group.

	BoA	RA	NFA	FM	LB	Consumers				
RM Options						All	Rural	Urban	Fem.	Male
*Business-as-usual*										
Option A	−0.39	−0.29	**0.72**	−0.14	−0.54	−0.19	−0.22	−0.16	−0.07	−0.23
Option B	−0.05	−0.01	−0.12	−0.10	−0.06	−0.05	−0.05	−0.04	**−0.03**	−0.05
Option C	**0.31**	**0.18**	−0.56	**−0.07**	**0.32**	**0.07**	**0.06**	**0.05**	−0.07	**0.10**
*Sustainable management*										
Option A	−0.34	−0.19	**0.52**	−0.12	−0.44	−0.19	−0.20	−0.16	−0.06	−0.23
Option B	0.09	0.11	−0.12	**0.23**	0.24	0.14	0.17	0.12	**0.15**	0.15
Option C	**0.38**	**0.20**	−0.44	0.20	**0.48**	**0.23**	**0.24**	**0.19**	0.08	**0.27**

The bold figures refer to the highest net flows, representing the dominant RM options for each stakeholder group (per scenario). *Abbreviations*: BoA, Board of Agriculture; RA, Regional authorities; NFA, National Food Agency; FM, Fishermen; LB, Local businesses; Option A, No derogation; Option B, Derogation plus consumer information; Option C, Derogation.

## Data Availability

Not applicable.
